# Linking humans and the environment in the spread of antimicrobial resistant *E. coli* in a rural community of South Africa. A One Health case study

**DOI:** 10.3389/fmicb.2026.1737700

**Published:** 2026-04-07

**Authors:** Solanka Ellen Ledwaba, Mpho Mphego, Natasha Potgieter

**Affiliations:** 1 Department of Biochemistry, Microbiology and Bioinformatics, Faculty of Science, Rhodes University, Makhanda, South Africa; 2 Department of Biochemistry and Microbiology, Faculty of Science, Engineering and Agriculture, University of Venda, Thohoyandou, South Africa

**Keywords:** antimicrobial resistance, *E. coli*, environment, humans, One Health, rural

## Abstract

Antimicrobial resistance is a global health crisis that threatens vulnerable populations in low-resource settings. Using the One Health approach, this study investigated AMR *in E. coli* among children under 5 years old and their surrounding living environments in Lwamondo village, South Africa. From 47 paired stool and soil samples, a total of 117 and 94 *E. coli* strains were isolated. Isolates were confirmed by PCR, and antimicrobial susceptibility testing was performed against commonly used antibiotics, followed by PCR for β-lactam resistance genes. Phenotypic β-lactam resistance was observed in children under 3 years of age (27–56%). Chloramphenicol was the most frequently detected antibiotic in both stool (41%) and soil (50%) isolates, followed by amoxicillin (27% in stool, 32% in soil). The *blaTEM* was the most predominant gene, detected in both the stools (36%) and soil (26%) isolates. Phylogenetic analysis revealed that the majority of AMR *E. coli* belong to Group A. The findings of this study demonstrate the interconnectedness between humans and their surrounding environment, which can both serve as important reservoirs for the transmission of AMR *E. coli*.

## Introduction

Antimicrobial resistance (AMR) is a persistent global health threat, estimated to have contributed to approximately 1.27 million deaths worldwide (Murray et al., 2022). Among bacterial pathogens driving this crisis, *E. coli* contributes significantly due to its adaptability across human, animal, and environmental reservoirs (World Health Organization, 2014). *E. coli* has been classified by WHO as a critical priority pathogen because of its capacity to develop resistance to clinically important antibiotics, including third-generation cephalosporins and carbapenems (Sati et al., 2025). The increasing prevalence of *E. coli* resistant strains poses a growing concern in low-resource settings, where surveillance and control measures are limited.

Children under the age of five are vulnerable to enteric infections due to their less developed immune systems and frequent exposure to contaminated environments, including poor hygiene practices and ingestion of contaminated soil during play, heightens exposure to pathogens (Kotloff et al., 2013; Ngure et al., 2013). Water and soil from outdoor playgrounds have been shown to harbor multiple pathogens simultaneously, suggesting that children who unintentionally ingest soil may be exposed to mixed doses of pathogens (Baker et al., 2018; Medgyesi et al., 2019). *E. coli* has been frequently reported to cause diarrheal diseases in children under the age of 5 years in the rural communities of Limpopo province in South Africa (Ledwaba et al., 2018; Potgieter et al., 2023). However, there is limited information on the occurrence and distribution of AMR *E. coli* in children and their immediate soil environment.

A One Health approach integrating human, animal, and environmental health is essential for understanding AMR transmission in rural, low-resource settings characterized by inadequate sanitation, close human-animal interactions, limited access to clean water and healthcare (Ngure et al., 2013; Karambwe et al., 2024). Environmental reservoirs, such as contaminated soil, also facilitate the transmission of microbes and are exacerbated by practices such as waste disposal and animal contact (Pickering et al., 2012; Fatoba et al., 2022). In certain rural areas of Limpopo province, reliance on pit toilets, use of untreated water for drinking and household purposes (Ledwaba et al., 2019), and the use of animal manure heighten the potential risks of acquiring diseases (Heuer et al., 2011). This study, therefore, aims to investigate the distribution of AMR profiles of *E. coli* isolated from children and their shared environment, and to assess the potential public health implications of these resistance patterns in the context of pathogen transmission between humans and the environment in rural settings.

## Materials and methods

### Study site and ethics

**FIGURE 1:**
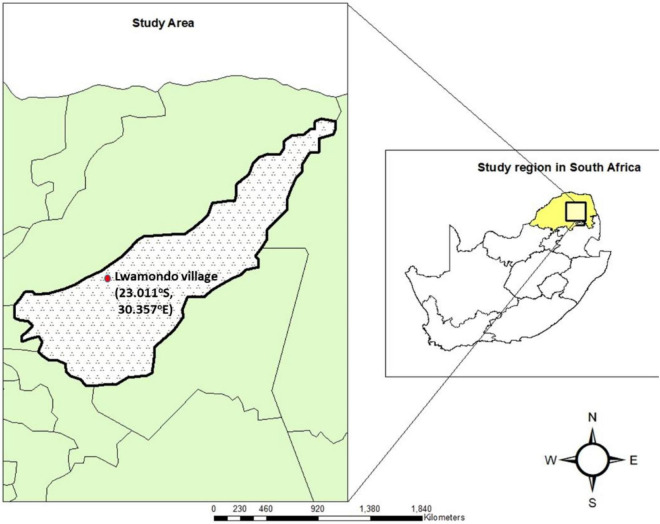
Geographic location of Lwamondo village in Limpopo Province, South Africa. The study site (23.011°S, 30.357°E) is indicated by a red marker. Limpopo Province is highlighted in yellow. The map was generated using ArcMap 10.5 (Environmental Systems Research Institute, United States), with administrative boundary shapefiles obtained from the Municipal Demarcation Board of South Africa and Statistics South Africa.

The study was conducted in Lwamondo village in the Vhembe District, Limpopo province, South Africa (Figure 1), a rural community of approximately 20,000 residents (Statistics South Africa, 2011). Ethical approval was granted by the University of Venda Research Ethics Committee (SMNS/20/MBY/11/0504). Informed consent was obtained from parents or guardians of the child < 5 years of age. A questionnaire was used to collect demographic data and information on each study participant’s living conditions.

### Inclusion and exclusion criteria

#### Inclusion criteria

Households within Lwamondo village with at least one child under 5 years were eligible. Participation was voluntary, and written informed consent was obtained from the parent or legal guardian prior to enrollment. One child per household was recruited and corresponding stool and soil samples were collected.

#### Exclusion criteria

Households without a child under the age of 5 years, or those in which informed consent was not provided, or households where an eligible child was absent at the time of sampling were excluded from the study.

### Sample collection

Sample collection was conducted from August 2022 to June 2023. Households were randomly selected to ensure a representative sample. Stool samples from 47 children under 5 years of age and soil samples were collected concurrently from their respective household play areas, constituting a paired household-level sampling design. All collected samples were stored in a cooler box with ice and processed within 4 h at the University of Venda laboratory for analysis.

### Bacterial isolation and Identification

Stool samples were enriched in peptone water and cultured on Eosin Methylene Blue agar (EMB) at 37°C for 24 h followed by subculturing on MacConkey agar at 37°C for 24 h to obtain pure colonies. Soil samples were sieved using a fine mesh sifter (Zhongbao Hardware Mesh Products Co, China) and a 1:50 dilution was prepared using phosphate buffer. A volume of 0.5 mL was poured on EMB (HiMedia^®^ Laboratories Pvt. Ltd., India) agar and spread using a sterile spreader and incubated for 24 h at 37°C. Thereafter, three isolates suspected to be *E. coli* were randomly selected from both stool and soil samples and cultured on MacConkey agar (Merck, Germany), and the plates were incubated overnight at 37°C (Garcia et al., 2011). Nutrient agar (HiMedia^®^ Laboratories Pvt. Ltd., India) was used to subculture isolates and obtain pure cultures. Presumptive *E. coli* isolates were confirmed using biochemical tests (catalase, urease, oxidase, triple sugar iron agar) according to the manufacturer’s instructions. Confirmed isolates were preserved in Brain Heart Infusion broth (Mast Diagnostics, United Kingdom) with 20% (v/v) glycerol and stored at −20°C.

To confirm presumptive *E. coli*, genomic DNA was extracted using the boiling method (Panchalingam et al., 2012). Molecular detection was performed using a T100 thermal cycler (Bio-Rad, United States). The *uidA* gene was amplified using the following primers: Forward: 5′-AAAACGGCAAGAAAAAGCAG-3′ and Reverse: 5′-ACGCGTGGTTAACAGTCTTGCG-3′ (Titilawo et al., 2015). PCR amplification was carried out in a total 25 μL reaction mixture containing 12.5 μL of 2 × Ampliqon Taq master mix red (Ampliqon, Denmark), 1 μL of 10 μM of each primer, nuclease-free water, and DNA template. PCR conditions consisted of an initial denaturation at 95°C for 5 min, followed by 35 cycles of denaturation at 95°C for 30 s, annealing at 58°C for 1 min, and extension at 72°C, with final extension at 72°C for 8 min. *E. coli* ATCC 25922 DNA served as the positive control. The PCR products were separated using 2% agarose gel stained with ethidium bromide and visualized using Omega Fluor Plus (Gel Imaging system, Vacutec^®^). The visual gel picture is shown in Supplementary Figure S1.

### Antibiotic susceptibility testing

The Kirby-Bauer disk diffusion method was used to assess antibiotic susceptibility against the following antibiotics: ampicillin (10 μg), amoxicillin (25 μg), cefotaxime (30 μg), cefoxitin (30 μg), cephalexin (30 μg), ciprofloxacin (5 μg), azithromycin (15 μg), aztreonam (30 μg), gentamicin (10 μg), amikacin (30 μg), tetracycline (30 μg), chloramphenicol (30 μg), and nalidixic acid (30 μg). The zone of clearance was assessed according to the Clinical and Laboratory Standards Institute (CLSI) guidelines (Clinical and Laboratory Standards Institute [CLSI], 2018). Isolates exhibiting resistance to two or more antibiotics were classified as multidrug resistant.

### Antimicrobial resistance gene detection

The extracted genomic DNA was used to further assess β-lactam resistance genes. Multiplex PCR was performed using *blaTEM* (Forward: 5′-GTCGCCGCATACACTATTCTCA-3′ and Reverse 5′-CGCTCGTCGTTTGGTATGG-3′), *blaSHV* (Forward: GCCTTGACCGCTGGGAAAC-3′ and Reverse 5′-GGCGTATCCCGCAGATAAAT-3′), and *blaCTX-M* (Forward: 5′-CGGGAGGCAGACTGGGTGT-3′ and Reverse: 5′-TCGGCTCGGTACGGTCGA-3′) primers (Cai et al., 2012). PCR amplification was carried out in a total 25 μL reaction mixture containing 12.5 μL of 2 × Qiagen multiplex PCR master mix (Qiagen, Germany), 0.5 μL of 10 μM of each primer, nuclease-free water, and DNA template. PCR conditions included 35 cycles of denaturation (95°C, 30 s), annealing (58°C, 1 min), and extension (72°C, 1 min). The 1.5% agarose gel electrophoresis, which was visualized in Omega Fluor Plus (Gel Imaging system, Vacutec^®^) was used to verify the amplification of the PCR products. The visual gel picture is shown in Supplementary Figure S2.

### Phylogenetic grouping

Phylogenetic groups (A, B1, B2, D) of *E. coli* were determined using multiplex PCR targeting *chuA* (Forward: 5′-GACGAACCAACGGTCAGGAT-3′ and Reverse: 5′-5′-TGCCGCCAGTACCAAAGACA-3′), *yjaA* (Forward: 5′-TGAAGTGTCAGGAGACGCTG-3′ and Reverse 5′-ATGGAGAATGCGTTCCTCAAC-3′), and *TSPE4.C2* (Forward: 5′-GAGTAATGTCGGGGCATTCA-3′ and Reverse: 5′-CGCGCCAACAAAGTATTACG-3′) primers (Clermont et al., 2000). PCR amplification was carried out in a total of 25 μL reaction mixture containing 12.5 μL of 2 x Ampliqon Taq master mix red (Ampliqon, Denmark), 1 μL of 20 μM of each primer, nuclease-free water, and DNA template. The cycling conditions for the PCR were 5 min at 95°C, 35 cycles of denaturation at 95°C for 40 s, annealing at 57°C for 40 s, an extension at 72°C for 40 s, and a final extension lasting 8 min at 72°C. *E. coli* ATCC 25922 served as the control strain. The visual gel picture is shown in Supplementary Figure S3.

### Data analysis

GraphPad Prism 8 (GraphPad Software) was used to generate the heatmap, PCA and the figures. The Principal Component Analysis was performed on a quantitative resistance matrix linking phenotypic antibiotic resistance frequencies to detected β-lactam resistance genes, with analyses grouped by sample source

## Results

### Demographic and sanitation characteristics

The 47 children (66% male, 34% female) had a mean age of 2.5 years (51% aged 0–2 years, 49% aged 3–5 years) (Table 1). Of these, 34% (16/47) reported prior diarrheal episodes, and 77% (36/47) had received antibiotics in the past 4 weeks. Breastfeeding was common in 70% (33/47) of cases, with 30% (14/47) receiving formula or mixed feeding. Sanitation was inadequate, with 77% (36/47) of households using pit toilets, 2% (1/47) using children’s toilets, and 21% (10/47) having flush toilets. Hand washing was limited (38% (18/47) after toilet use and 11% (*n* = 5/47) washed hands before meals. Child-animal contact was reported in 85% (40/47) of households (e.g., chickens, goats), and 100% (47/47) of the households used animal stools as a source of manure, increasing environmental contamination risks.

**TABLE 1:** Demographic and sanitation characteristics.

Characteristic	Description	N (%)
Sex	Female	16 (34%)
	Male	31 (66%)
Illness in the past 4 weeks	Diarrhea	16 (34%)
	No illness	31 (66%)
Antibiotic use	Children who received antibiotics	36 (77%)
	Children who did not receive antibiotics	11 (23%)
Feeding practices	Breastfeeding	33 (70%)
	Formula or mixed feeding	14 (30%)
Sanitation facilities	Pit toilets	36 (77%)
	Children’s toilets	1 (2%)
	Flush toilets	10 (21%)
Water sources	Standpipes	31 (66%)
	Boreholes	12 (26%)
	Rivers	4 (9%)
Water treatment	Households treating water before use	21 (45%)
Waste disposal practices	Yard disposal	25 (53%)
	Municipal collection	10 (21%)
	Open disposal in the field	12 (25%)
Hand washing practices	After toilet use	18 (38%)
	Before meals	5 (11%)
Child-animal contact	Households reporting contact (e.g., chickens, goats)	40 (85%)
	No contact reported	7 (15%)
Animal manure use	Households using animal stools as manure	47 (100%)

### Distribution of antibiotic resistance genes across the age group

**FIGURE 2:**
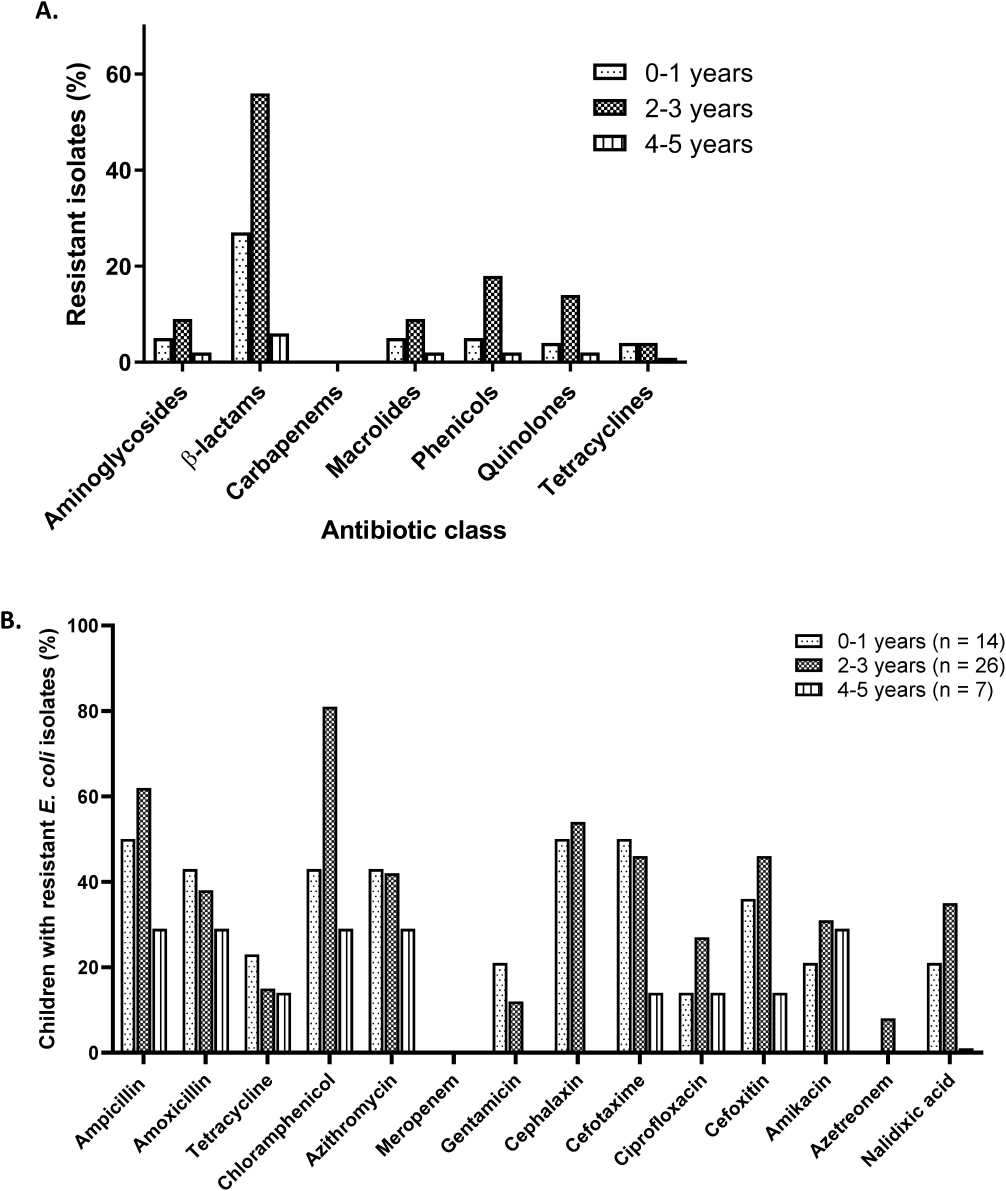
Antibiotic distribution of *E. coli* by age group. **(A)** Antibiotic resistance summarized by antibiotic class, expressed as percentage of *E. coli* isolates within each age group (*n* = 117). **(B)** Resistance to individual antibiotics tested, expressed as the percentage of the number of children within each group with resistant *E. coli* isolates.

A total of 47 children were enrolled in the study, from whom 117 *E. coli* isolates were recovered from stool samples. The resistance prevalence in Figure 2A was calculated based on the total number of *E. coli* isolates recovered within each group. Overall, β-lactam resistance was predominantly high in children aged between 0–1 and 2–3 years (27–56%). At the child level (Figure 2B), resistance prevalence was calculated as the proportion of children in each group with resistant *E. coli* isolates. The highest resistance was observed for chloramphenicol, detected among children aged 2–3 years [81% (21/26)]. Gentamicin was observed only in children aged 0–1 years (21%, 3/14) and 2–3 years (12%, 3/26), whereas aztreonam was detected only in children aged 2–3 years (8%, 2/26).

### Antibiotic resistance profiles

Antibiotic susceptibility testing was conducted on 211 confirmed *E. coli* isolates (stools and soil) against 14 antibiotics (Table 2). In stool samples, the highest phenotypic resistance was observed for chloramphenicol (41%, 48/117), cefotaxime (30%, 35/117), and cephalexin (29%, 34/117). The lowest resistance was noted for aztreonam (2%, 2/117), gentamicin (4%, 5/117), and tetracycline (9%, 11/117). In soil samples, chloramphenicol exhibited the highest resistance (50%, 47/94), followed by ampicillin (40%, 38/94) and azithromycin (37%, 35/94). The lowest resistance was observed for aztreonam (3%, 3/94), nalidixic acid (6%, 6/94), and ciprofloxacin (10%, 9/94). No isolates were resistant to meropenem.

**TABLE 2:** Phenotypic antibiotic resistance of *E. coli* isolates from stool and soil samples.

Antibiotic	Stool *n* = 117 (%)	Soil *n* = 94 (%)
Ampicillin	18 (15%)	38 (40%)
Amoxicillin	31 (27%)	30 (32%)
Tetracycline	11 (9%)	7 (7%)
Chloramphenicol	48 (41%)	47 (50%)
Azithromycin	26 (22%)	35 (37%)
Meropenem	0 (0%)	0 (0%)
Gentamicin	5 (4%)	11 (12%)
Cephalexin	34 (29%)	31 (33%)
Cefotaxime	35 (30%)	19 (20%)
Ciprofloxacin	13 (11%)	9 (10%)
Cefoxitin	29 (25%)	31 (33%)
Amikacin	18 (15%)	10 (11%)
Aztreonam	2 (2%)	3 (3%)
Nalidixic acid	20 (17%)	6 (6%)

### Multidrug resistance

**FIGURE 3:**
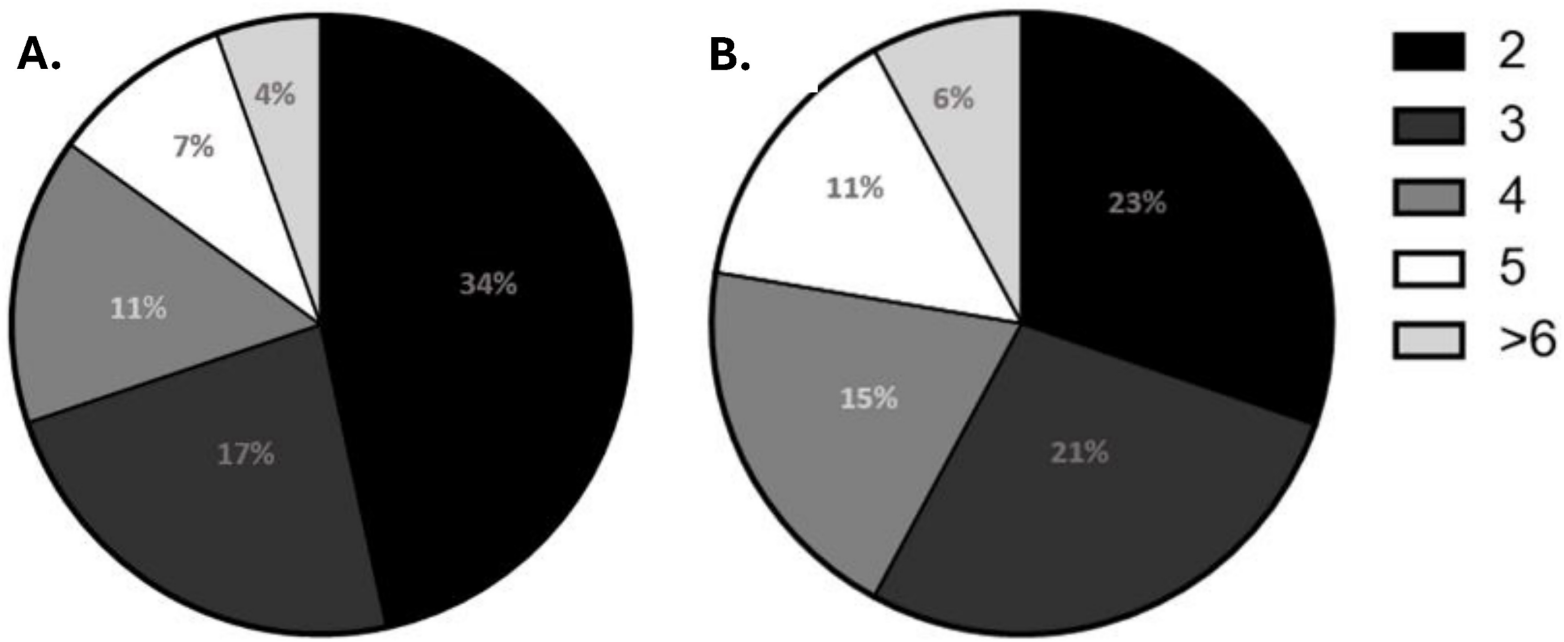
Frequency of phenotypic multidrug resistance combinations among *E. coli* isolates recovered from **(A)** stool and **(B)** soil isolates. Pie chart segments represent the proportion (%) of isolates resistant to two or more antibiotics.

Overall, MDR was observed in 75% (88/117) of the stool isolates and 88% (83/94) of the soil isolates (Supplementary Tables 1,2). In stool samples, 34% (40/117) of isolates were resistant to two antibiotics simultaneously (Figure 3), with the most frequent combination being ampicillin and cephalexin (3%, 4/117) (Supplementary Table 1). The combination of nalidixic acid, amikacin, and chloramphenicol was observed in 2% (2/117) of isolates. In soil samples, the most common two-antibiotic combination was cephalexin and chloramphenicol (3%, 3/94). Visual phenotypic MDR profiles of some of the resistant isolates are shown in Supplementary Figure S4.

### Detection of β-lactam resistance genes

The presence of β-lactam resistance genes was assessed in all confirmed *E. coli* isolates (Table 3). Of the three genes tested, *blaTEM* was the predominant resistance gene (33%, 70/211) across both sample types. In stool samples, the *blaTEM* was detected in 39% (46/117), *blaSHV* in 7% (8/117), and *blaCTX-M* in 3% (4/117). In soil samples, *blaTEM* was detected in 26% (24/94), *blaSHV* in 2% (2/94), and no *blaCTX-M* genes were detected. Combinations of resistance genes were observed (Table 3). In stool samples, 2% (2/117) of isolates harbored *blaTEM* + *blaSHV* + *blaCTX-M*, 3% (3/117) had *blaSHV* + *blaTEM*, and 2% (2/117) had *blaTEM* + *blaCTX-M* combinations. In soil samples, only *blaSHV* + *blaTEM* combinations were detected in 1% (1/94) of isolates.

**TABLE 3:** Distribution of β-lactam resistance genes and gene combinations among *E. coli* isolates from stool and soil.

Antibiotic resistance genes	Stool *n* = 117 (%)	Soil *n* = 94 (%)
*blaCTX-M*	4 (3%)	0
*blaTEM*	46 (39%)	24 (26%)
*blaSHV*	8 (7%)	2 (2%)
Multiple resistance		
*blaTEM* + *blaSHV* + *blaCTX-M*	2 (2%)	0 (0%)
*blaSHV* + *blaTEM*	3 (3%)	1 (1%)
*blaTEM* + *blaCTX-M*	2 (2%)	0 (0%)

### Distribution of antibiotic resistance genes and antibiotic resistance

**FIGURE 4:**
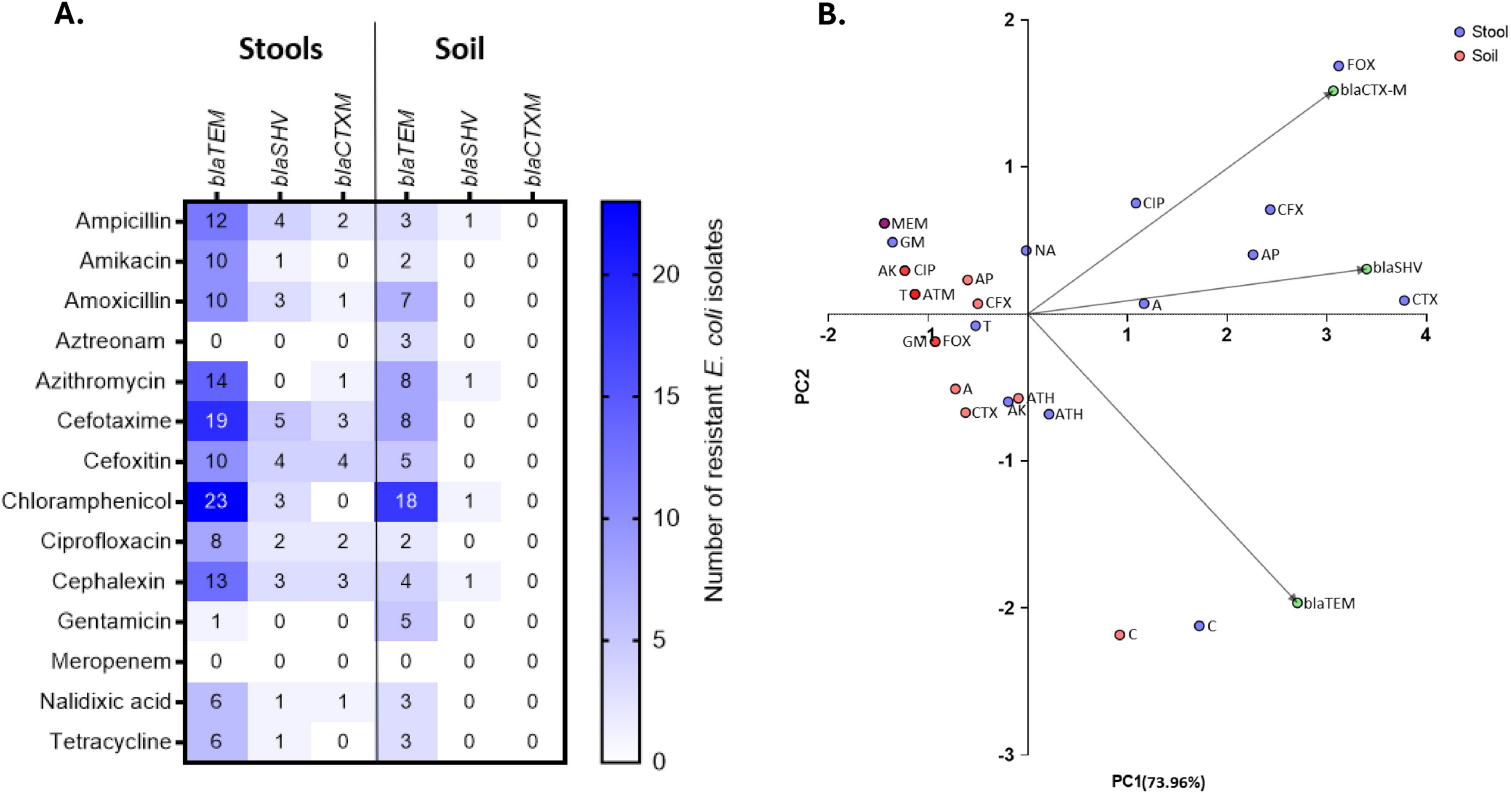
Comparison of antibiotic patterns in stools and soil isolates. **(A)** Heatmap showing raw counts of phenotypically resistant *E. coli* isolates exhibiting β-lactam resistance profiles among the stool (*n* = 117) and soil (*n* = 94) isolates. Cell values represent the number of resistant isolates. Each row represents an antibiotic tested, and each column represents β-lactam-positive *E. coli* isolates. **(B)** The principal component analysis (PCA) illustrating the relationship between phenotypic antibiotic and β-lactam resistance genes among stools and soil isolates. PCA constructed using a quantitative matrix in which rows represent individual antibiotics, columns represent resistance genes and cell values correspond to the percentage of resistant isolates. Points are colored by sample source (stool or soil) and arrows indicate resistance loadings. Keywords: A, amoxicillin; AK, amikacin; AP, ampicillin; ATH, azithromycin; ATM, aztreonam; C, chloramphenicol; CFX, cephalexin; CIP, ciprofloxacin; CTX, cefotaxime; FOX, cefoxitin; GM, gentamicin; MEM, meropenem; NA, nalidixic acid; T, tetracycline.

The comparison of phenotypic and genetic resistance across the stools and soil isolates were further assessed using the heatmap (Figure 4A). The stool isolates displayed broad resistance across multiple antibiotic classes of β-lactams, and this distribution was genotypically consistent with the presence of *blaTEM* and *blaSHV*. The soil isolates showed lower overall resistance with high chloramphenicol and *blaTEM*. To explore the association between phenotypic and genotypic patterns between the stools and soil isolates the PCA plot was assessed (Figure 4B). The stool isolates clustered mainly in PC1, driven by *blaSHV* and *blaTEM*, while soil isolates clustered resistance determinants. The soil isolates showed limited phenotypic and genetic resistance.

### Phylogenetic grouping of *E. coli* isolates

Multiplex PCR classified 75 *E. coli* isolates harboring antibiotic resistance genes into phylogenetic groups A, B1, B2, and D (Table 4). In stool samples, 81% (38/47) belonged to group A, 2% (1/47) to group B1, and 21% (10/47) to group D. In soil samples, 96% (27/28) were classified as group A, with no isolates in groups B1, B2, or D detected.

**TABLE 4:** Distribution of phylogenetic groups among *E. coli* isolates positive for β-lactam resistance genes from stool and soil samples.

Phylogroup	Stool *n* = 47 (%)	Soil *n* = 28 (%)	Total *n* = 75 (%)
A	38 (81%)	27 (96%)	65 (87%)
B1	1 (2%)	0 (0%)	1 (1.33%)
B2	0 (0%)	0 (0%)	0 (0%)
D	10 (21%)	0 (0%)	10 (13%)

## Discussion

This study investigated the distribution of AMR *E. coli* in children and their surrounding environment in the Lwamondo village, a rural community where livestock herding is widely practiced. The One Health approach was integrated into the study to assess the interconnectedness between children and the environment using soil as a medium of exposure to AMR *E. coli*. A paired sampling method was utilized in this study to assess the community-level distribution of AMR *E. coli*, enabling direct comparison of resistance patterns within shared exposure settings, in contrast to conventional AMR surveillance studies that examine clinical or environmental samples independently.

The antibiotic with the highest resistance detected was chloramphenicol (41% in stools and 50% in soils). Similar resistance to chloramphenicol has been reported in the environment from agricultural soils in South Africa, where values exceed 60% (Fatoba et al., 2022), and in 36% of stool isolates in Iraq (Al-Saadi et al., 2018). Chloramphenicol is a broad-spectrum antibiotic and its use in South Africa is mostly limited to ophthalmic applications and severe bacterial infections. The high levels of chloramphenicol resistance observed, particularly in soil, are therefore unlikely to reflect ongoing widespread use, but instead may be attributed to historical selection and co-selection of resistance determinants that persist in environmental reservoirs. The increase in antibiotic resistance to β-lactams in this study also raises concern. Soil isolates showed considerably higher resistance to ampicillin (40%) than in stools (15%), while amoxicillin was moderate across the isolates (27–32%). These findings are comparable to those of environmental isolates in soil (Binh et al., 2007) and stool-based studies in children (Seidman et al., 2009), which report increased amoxicillin resistance. The moderate distribution levels of antibiotics observed in the environment may therefore reflect less intensive agriculture antibiotic use compared to highly commercialized systems (Ager et al., 2023).

One of the factors that could have contributed to the elevated AMR in soil in the study setting is the use of animal manure as fertilizer. Various studies have demonstrated that manure from livestock farms carries antibiotic residues that can persist and negatively impact the soil microbiome (Fatoba et al., 2022; Marutescu et al., 2022). MDR was observed with the highest phenotypic resistance with 2–4 combinations in both stools (34–11%) and soil (23–15%) isolates. These MDR combinations are prevalent (Barreto et al., 2009; Huang et al., 2018), and this poses a health risk to children, making it difficult to treat AMR infections. This increased MDR combinations reinforces the interconnectedness of humans and the environment, with soil as a potential reservoir of AMR *E. coli*.

When *E. coli* resistance was analyzed by age group, children aged < 3 years exhibited a higher burden than older children. Overall, the β-lactam resistance was more common in this age group. These findings are consistent with reports from the WHO, which have identified extended-spectrum β-lactamase (ESBL)-*E. coli* resistance globally as a priority pathogen of concern (Sati et al., 2025). The β-lactam resistance in children < 1 year was evident with elevated levels of amoxicillin (43%), ampicillin (50%), cephalexin (50%), cefotaxime (50%) and cefoxitin (36%). The high prevalence of β-lactam resistance is alarming since these antibiotics are also used in veterinary and agricultural settings (Mesa et al., 2006). Given that β-lactams are used as first-line treatment, this further suggests that resistance to β-lactams is not only widespread; it is also amplified through various systems (Sahoo et al., 2012; Mapanguy et al., 2021; Milenkov et al., 2024).

The most frequently detected β-lactamase gene was *blaTEM* in both stool (39%) and soil (26%) isolates, followed by the *blaSHV* gene (7, 2%). The presence of these genes indicates potential horizontal gene transfer (Mishra and Lercher, 2025). Other studies in Iran (Afsharikhah et al., 2023), Sudan (Dirar et al., 2020), and Egypt (Mohamed et al., 2020) have also reported on increased distribution of β-lactamase resistance. The distribution of β-lactam genes and phenotypic resistance, as determined by heatmap analysis, further illustrated the spread of resistance across both stool and soil isolates. Clustering of β-lactam genes was observed mainly in stool isolates, suggesting the presence of β-lactam genes associated with commonly used antibiotics. These combinations raise a concern, with the rise in MDR strains that persist in both children and the environment. Although the distribution of β-lactam genes was observed throughout, soil isolates exhibited a wider variation in their resistance profiles. This variability reflects multiple environmental sources of contamination that could have contributed to the diverse selective pressures (Gurmessa et al., 2021; Fatoba et al., 2022; Hasib et al., 2024; Zhao et al., 2025).

The stool isolates, on the other hand, displayed similar resistance profiles, thereby reflecting the influence of comparable clinical antibiotic pressure. Although conventional PCR confirmed the presence of β-lactam-resistant genes, it does not assess gene expression. Further studies using qRT-PCR may help clarify active resistance mechanisms. *E. coli* phylogenetic grouping provides insight into ecological origin and virulence potential. Groups A and B1 are typically associated with commensal strains, while B2 and D are associated with pathogenic linages (Clermont et al., 2000). Most of the isolates belonged to group A (87%), which is typically associated with commensal strains. The pathogenic phylogroup D was detected only in stool samples (21%). This distribution suggests that while commensal *E. coli* strains dominate both clinical and environmental samples, they also play an important role in the dissemination of AMR, contributing to additional health risks to humans.

Children under the age of 5 years have not yet mastered good hygiene practices and are vulnerable (Rah et al., 2015), and this phenomenon was evident in the findings of this study. The use of pit latrines is common in rural communities (Beukes et al., 2017; Ledwaba et al., 2019; Smith et al., 2023), with 76% observed in the study setting. Although pit latrines offer convenient sanitation in areas where wastewater treatment plants are not put into place, they also act as key reservoirs that collect, retain, and release pollutants into the environment; and this includes infectious microbes, pharmaceutical products such as antibiotic derivatives, and disease-carrying vectors such as rodents and houseflies (Gwenzi et al., 2023).

Subsistence farming in the rural communities of Lwamondo village was prevalent, and the animals were reared in the yard where children played. This increases the potential risk of zoonotic disease contamination as the children can unintentionally ingest fecal material from fomites. Children in this region were previously reported to also be at risk of contamination of fecal material through handling of toys by children (Ledwaba et al., 2019). Improper waste disposal increases the chance of pathogen contamination and dissemination. The use of untreated animal manure to enrich agricultural soils is a norm in these settings; however, this practice has been recognized as a significant reservoir of enteric antimicrobial resistant bacteria posing a risk of environmental contamination (Johnson et al., 2016).

Similar environmental exposure dynamics have been reported in sub-Saharan Africa, where fecal contamination of the household environment has been associated with enteric pathogen detection in exposed populations (Holcomb et al., 2025). While that study focused on pathogen transmission, our findings extend this understanding by characterizing AMR profiles with environmental isolates. Although direct transmission pathways were not established in this study, the observed findings overlap in phenotypic and genotypic resistance patterns, suggesting the potential circulation of AMR *E. coli* between children and the environment. Children can introduce resistant strains into the soil through fecal contamination and poor hygiene or sanitation practices, while the soil acts as an environmental reservoir of resistant strains which can, in turn, serve as a source of exposure to children through play or ingestion. Soil contaminated with AMR *E. coli* poses not only a health risk to children but also serves as a long-term environmental reservoir that facilitates the persistence and spread of resistance among community members. These findings address AMR distribution using the One Health framework, highlighting the need for effective AMR surveillance across different settings (Ruckert et al., 2024).

## Conclusion

This study demonstrated that children’s stools and their shared environment (soil) serve as reservoirs of AMR *E. coli*. The β-lactam was prevalent across the age groups, and this was supported by the presence of β-lactam genes. Children under the age of 1 year carried the greatest burden, reflecting increased vulnerability to environmental exposure. Overall, the findings demonstrate that AMR affects humans and the environment both directly and indirectly, underscoring the need for integrated One Health strategies to strengthen antibiotic stewardship.

## Data Availability

The original contributions presented in the study are included in the article/Supplementary material, further inquiries can be directed to the corresponding author.
