# Supplementary material for: Linking humans and the environment in the spread of antimicrobial resistant E. coli in a rural community of South Africa. A One Health case study
Source: Front Microbiol. 2026 Apr 7;17:1737700. doi: 10.3389/fmicb.2026.1737700 (PMC13095841; doi:10.3389/fmicb.2026.1737700)
Supplement: Supplementary file 1 [file Data_Sheet_1.pdf]

**Linking humans and the environment in the spread of antimicrobial resistant *E. coli* in a rural community of South Africa. A One Health case study**

Solanka Ellen Ledwaba[1], Mpho Mphego[2], and Natasha Potgieter[2]

[1]Department of Biochemistry, Microbiology and Bioinformatics, Faculty of science, Rhodes University, Makhanda, South Africa.

[2]Department of Biochemistry and Microbiology, Faculty of Science, Engineering and Agriculture, University of Venda, Thohoyandou, South Africa

**Supplementary File**

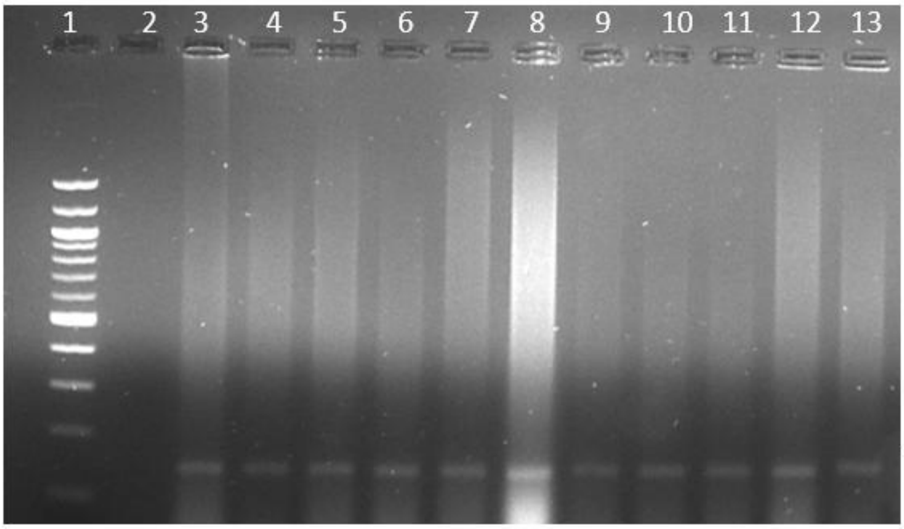


**Figure S1:** Gel electrophoresis image of the confirmed *E. coli* isolates using the *uidA* gene (147bp). L1: DNA ladder, L2: negative control, L3: positive control (*E. coli* 25922), L 4-13: PCR products obtained from isolates

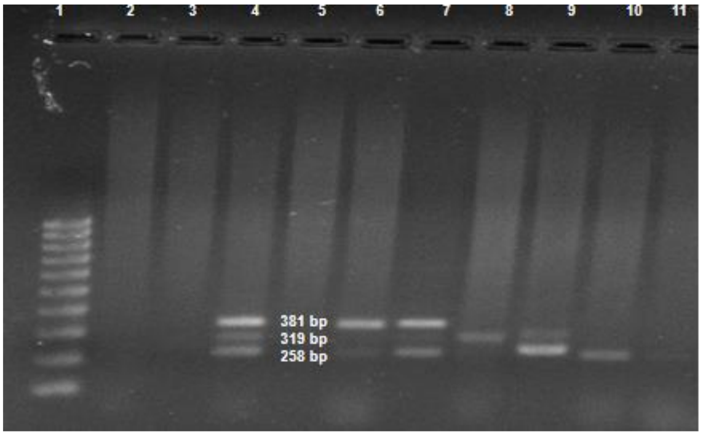


**Figure S2:** A gel electrophoresis of beta-lactam resistance genes m-PCR (*blaTEM* 258 bp, *blaSHV* 319 bp, *blaCTX-M* 381 bp). From left to right: L1: DNA ladder (1000 bp), L2: negative control, L3-11 PCR products obtained from isolates.

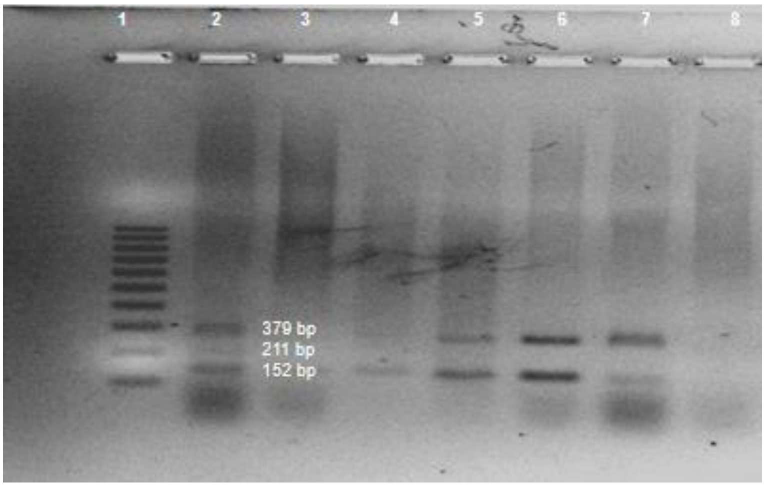


**Figure S3:** Gel electrophoresis of the multiplex PCR for *E. coli* phylogenetic grouping. L1: DNA ladder (1000 bp); L2: positive control group B2 (*TspE4c*2 152 bp, *yjaA*.1211 bp, *chuA* 279 bp); L3-L7 tested isolates and L8: negative control.

**Supplementary Table 1: Phenotypic multidrug resistance combinations observed in stool isolates of children.**

| Antibiotic combination | Number of *E. coli* isolates |
|---|---|
| **Resistance to two antibiotics)** | **n=%** |
| **FOX + CIP** | 1 (0.85%) |
| **ATH + AP** | 1 (0.85%) |
| **AP + CIP** | 1 (0.85% |
| **ATH + FOX** | 1 (0.85%) |
| **ATH + NA** | 2 (1.71%) |

| | |
|---|---|
| **AP + T** | 1 (0.85%) |
| **AP +CFX** | 4 (3.42%) |
| **AP + C** | 3 (3.42%) |
| **AK + ATH** | 1 (0.85%) |
| **C + NA** | 2 (1.71%) |
| **C + CTX** | 2 (1.71%) |
| **C+CFX** | 1 (0.85%) |
| **A+C** | 1 (0.85%) |
| **FOX +T** | 1 (0.85%) |
| **AP+CTX** | 1 (0.85%) |
| **A+T** | 1 (0.85%) |
| **C+A** | 3 (3.42%) |
| **C+FOX** | 1 (0.85%) |
| **FOX+GM** | 1 (0.85%) |
| **AP+AK** | 1 (0.85%) |
| **CIP+AK** | 1 (0.85%) |
| **CIP+C** | 1 (0.85%) |
| **AP+ A** | 1 (0.85%) |
| **CTX+A** | 1 (0.85%) |
| **CTX+ATH** | 1 (0.85%) |
| **C+AK** | 1 (0.85%) |
| **NA+A** | 1 (0.85%) |
| **ATH+A** | 1 (0.85%) |
| **NA+C** | 2 (1.71%) |
| **Total** | **40 (34.18%)** |
| **Resistance to three or more antibiotics)** | |
| **CFX+CTX+FOX** | 1 (0.85%) |
| **CTX+AP+C** | 1 (0.85%) |
| **ATH+CFX+A** | 1 (0.85%) |
| **FOX+C+CIP** | 1 (0.85%) |
| **CIP+CFX+ATH** | 1 (0.85%) |
| **ATH+C+AP** | 1 (0.85%) |
| **CFX+CTX+A** | 1 (0.85%) |
| **NA+AK+C** | 2 (1.71%) |
| **C+AK+A** | 1 (0.85%) |
| **GM+ATH+CFX** | 1 (0.85%) |
| **AP+T+C** | 1 (0.85%) |
| **NA+CIP+C** | 1 (0.85%) |
| **FOX+AP+ATH** | 1 (0.85%) |
| **ATM+C+AP** | 1 (0.85%) |
| **ATH+A+CTX** | 1 (0.85%) |
| **AK+C+CTX** | 1 (0.85%) |
| **AK+C+FOX** | 1 (0.85%) |

| | |
|---|---|
| **C+T+NA+CFX** | 1 (0.85%) |
| **NA+AP+FOX** | 1 (0.85%) |
| **FOX+CTX+AP+CIP+A+NA+CFX+ATH** | 1 (0.85%) |
| **FOX+CTX+AP+CFX** | 1 (0.85%) |
| **FOX+CTX+AP+C+A+NA+CFX** | 1 (0.85%) |
| **FOX+CTX+AP+C+A+CFX** | 1 (0.85%) |
| **FOX+CTX+C+A+CFX** | 1 (0.85%) |
| **CTX+AP+A+NA+CFX** | 1 (0.85%) |
| **AP+C+A+CFX** | 1 (0.85%) |
| **CTX+AK+T+ATH** | 1 (0.85%) |
| **CTX+AP+A+NA+CFX+ATH** | 1(0.85%) |
| **ATH+ATM+NA+A+C+GM+AP+CTX** | 1 (0.85%) |
| **ATH+CFX+C+A** | 1 (0.85%) |
| **FOX+AK+A+CFX** | 1 (0.85%) |
| **ATH+A+AP+CTX+FOX** | 1 (0.85%) |
| **CTX+C+T+CFX** | 1 (0.85%) |
| **A+T+AK+C** | 1 (0.85%) |
| **FOX+CIP+C+CFX** | 1 (0.85%) |
| **FOX+AP+C+A+CFX** | 1 (0.85%) |
| **CTX+AP+C+A+CFX** | 1 (0.85%) |
| **AP+GM+C+CIP** | 1 (0.85%) |
| **FOX+AP+C+CFX** | 1 (0.85%) |
| **CTX+A+NA+CFX** | 1 (0.85%) |
| **FOX+C+T+A+NA** | 1 (0.85%) |
| **GM+NA+CFX+ATH** | 1 (0.85%) |
| **CTX+AP+CFX+ATH** | 1 (0.85%) |
| **CTX+AP+AK+NA+ATH** | 1 (0.85%) |
| **FOX+CTX+ATH+CFX+NA** | 1 (0.85%) |
| **Total** | **48 (41.02%)** |
| **Overall total** | **88 (75.20%)** |

Keywords: AP= Ampicillin, A= Amoxicillin, T= Tetracycline, C=Chloramphenicol, ATH= Azithromycin, GM= Gentamicin, CFX= Cephalexin, CTX= Cefotaxime, CIP= Ciprofloxacin, FOX= Cefoxitin, AK= Amikacin, ATM= Aztreonam and NA= Nalidixic acid.

## Supplementary Table 2: Phenotypic multidrug resistance combinations observed in soil isolates.

| **Antibiotic resistance combination** | **Number of *E. coli* isolates** |
|---|---|
| **Resistance to two antibiotics** | **n (%)** |
| **CFX+FOX** | 2 (2.12%) |
| **CFX+AK** | 1 (1.06%) |
| **T+ATH** | 2 (2.12%) |
| **CFX+ATH** | 1 (1.06%) |

| | |
|---|---|
| **AP+A** | 1 (1.06%) |
| **ATH+C** | 2 (2.12%) |
| **CFX+C** | 3 (3.19%) |
| **C+AK** | 1 (1.06%) |
| **AK+A** | 1 (1.06%) |
| **GM+C** | 1 (1.06%) |
| **CTX+C** | 1 (1.06%) |
| **C+T** | 1 (1.06%) |
| **FOX+T** | 1 (1.06%) |
| **FOX+ATH** | 1 (1.06%) |
| **CTX+CFX** | 1 (1.06%) |
| **AP+C** | 1 (1.06%) |
| **A+CFX** | 1 (1.06%) |
| **Total** | **22 (23%)** |
| **Resistance to three or more antibiotics** | |
| **FOX+CFX+ATH** | 2 (2.12%) |
| **FOX+C+CFX** | 2 (2.12%) |
| **AP+A+ATH** | 1 (1.06%) |
| **CIP+CFX+ATH** | 2 (2.12%) |
| **ATH+C+A** | 2 (2.12%) |
| **CTX+AP+ATH** | 1 (06%) |
| **CTX+CFX+ATH** | 2 (2.12%) |
| **C+CIP+ATH** | 1 (1.06%) |
| **GM+A+CFX** | 1 (1.06%) |
| **CTX+C+ATH** | 1 (1.06%) |
| **FOX+C+ATH** | 1 (1.06%) |
| **FOX+C+GM** | 1 (1.06%) |
| **FOX+T+ATH** | 1 (1.06%) |
| **C+CFX+ATH** | 1 (1.06%) |
| **FOX+T+CFX** | 1 (1.06%) |
| **CTX+CIP+AP** | 1 (1.06%) |
| **FOX+CTX+A+NA** | 1 (1.06%) |
| **GM+C+CFX+ATH** | 1 (1.06%) |
| **FOX+CTX+A+C** | 1 (1.06%) |
| **C+AP+CIP+A** | 1 (1.06%) |
| **FOX+C+AK+T** | 1 (1.06%) |
| **CTX+CIP+T+NA+ATH** | 1 (1.06%) |
| **CTX+GM+C+T+ATH** | 1 (1.06%) |
| **A+C+GM+AP** | 1 (1.06%) |
| **ATM+C+GM+CTX+FOX** | 1 (1.06%) |
| **NA+AK+C+GM+AP+FOX** | 1 (1.06%) |
| **FOX+AP+C+CIP+A+ATH** | 1 (1.06%) |

| | |
|---|---|
| **FOX+CTX+C+CIP+T+A+CFX** | 1 (1.06%) |
| **FOX+CTX+C+CIP+A+ATH** | 1 (1.06%) |
| **CTX+A+NA+ATM+FOX** | 1 (1.06%) |
| **FOX+CTX+AP+NA+A+ATH** | 1 (1.06%) |
| **FOX+CTX+C+CIP+NA** | 1 (1.06%) |
| **FOX+CTX+AK+A+CIP+C** | 1 (1.06%) |
| **CTX+C+A+ATH** | 1(1.06%) |
| **C+CIP+T+CFX+A** | 1 (1.06%) |
| **GM+C+NA+ATH** | 1 (1.06%) |
| **C+AP+A+ATH** | 1 (1.06%) |
| **FOX+AP+A+CFX+ATH** | 1 (1.06%) |
| **FOX+C+CFX+ATH** | 1 (1.06%) |
| **AP+A+CFX+ATH** | 1 (1.06%) |
| **CFX+C+A+ATH** | 1 (1.06%) |
| **FOX+CTX+GM+CFX** | 1 (1.06%) |
| **FOX+CFX+A+T+AP** | 2 (2.12%) |
| **FOX+AP+C+CFX** | 1 (1.06%) |
| **FOX+C+AK+A+CFX** | 1 (1.06%) |
| **Total** | **61 (65%)** |
| **Overall total** | **83 (88%)** |

Keywords: AP= Ampicillin, A= Amoxicillin, T= Tetracycline, C=Chloramphenicol, ATH= Azithromycin, , GM= Gentamicin, CFX= Cephalexin, CTX= Cefotaxime, CIP= Ciprofloxacin, FOX= Cefoxitin, AK= Amikacin, ATM= Aztreonam and NA= Nalidixic acid.

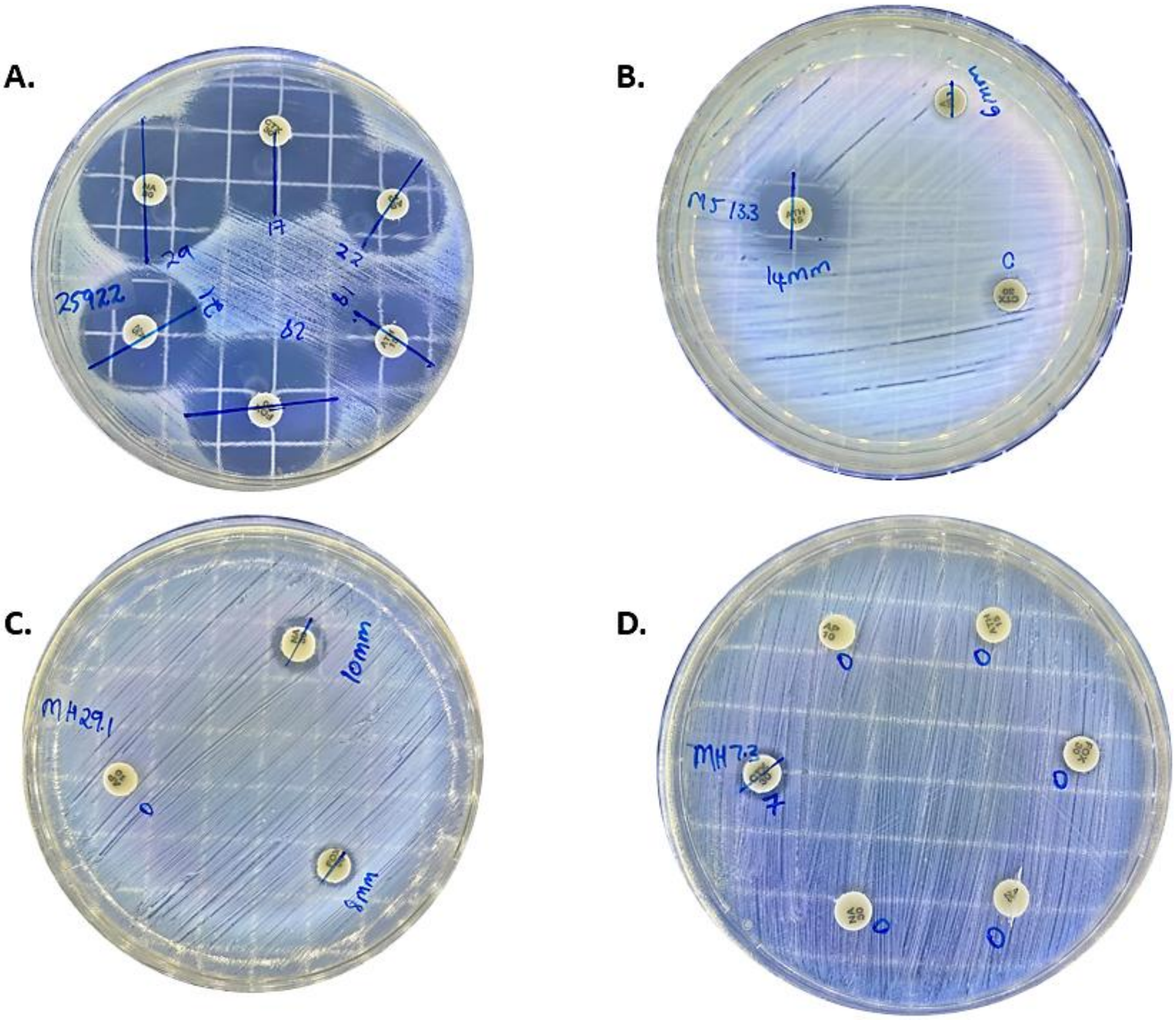


**Figure S4**: Antibiotic susceptibility testing performed using the Kirby Bauer disk diffusion method on Muller Hinton agar. Zone diameters were interpreted according to CLSI (2018) guidelines. **A.** Positive control (*E. coli* 25922) showing sensitivity to all tested antibiotics **B.** Isolate resistant to two antibiotics: amoxycillin (A), cefotaxime (CTX) and sensitive to azithromycin (ATH) **C.** Isolate resistant to three antibiotics: nalidixic acid (NA), ampicillin (AP) and cefoxitin (FOX). **D** Isolate resistant to 6 antibiotics tested: cefotaxime (CTX), ampicillin (AP), amoxycillin (A), nalidixic acid (NA), cefotaxime (CFX), and azithromycin (ATH). Values on the plates are represented in millimetres. In cases where zone edges partially overlapped with the adjacent inhibition zones, diameters were measured across the clearly defined axis of inhibition, and the full zone diameter was recorded in millimetres according to CLSI guidelines. Keywords: AP= ampicillin, A= amoxicillin, ATH= azithromycin CTX= cefotaxime, FOX= cefoxitin, NA= nalidixic acid.
